# Differential S-phase progression after irradiation of p53 functional versus non-functional tumour cells

**DOI:** 10.2478/raon-2014-0032

**Published:** 2014-11-05

**Authors:** Friedo Zölzer, Tamare Mußfeldt, Christian Streffer

**Affiliations:** 1 Institute of Medical Radiobiology, Medical Faculty, University Duisburg-Essen, Germany; 2 Department of Radiology, Toxicology and Civil Protection, Faculty of Health and Social Studies, University of South Bohemia in České Budějovice, Czech Republic

**Keywords:** x-rays, intra-S-phase checkpoint, flow cytometry, relative movement

## Abstract

**Background:**

Many pathways seem to be involved in the regulation of the intra-S-phase checkpoint after exposure to ionizing radiation, but the role of p53 has proven to be rather elusive. Here we have a closer look at the progression of irradiated cells through S-phase in dependence of their p53 status.

**Materials and methods.:**

Three pairs of tumour cell lines were used, each consisting of one p53 functional and one p53 non-functional line. Cells were labelled with bromodeoxyuridine(BrdU) immediately after irradiation, they were then incubated in label-free medium, and at different times afterwards their position within the S-phase was determined by means of flow cytometry.

**Results:**

While in the p53 deficient cells progression through S-phase was slowed significantly over at least a few hours, it was halted for just about an hour in the p53 proficient cells and then proceeded without further delay or even at a slightly accelerated pace.

**Conclusions:**

It is clear from the experiments presented here that p53 does play a role for the progress of cells through the S-phase after X-ray exposure, but the exact mechanisms by which replicon initiation and elongation is controlled in irradiated cells remain to be elucidated.

## Introduction

The crucial importance of the tumour suppressor protein p53 for the regulation of cell cycle progression after irradiation has been known for more than two decades. It was the papers of Kastan *et al*.^1^ and Kuerbitz *et al*.^2^ at the beginning of the 1990s that alerted radiation biologists to the fact that the G_1_-checkpoint was under the control of p53. The checkpoint itself, *i.e.* a radiation induced block of cell cycle progression before the entry into the S-phase had already been described in 1953 by Howard and Pelc^3^ for plant cells, and 15 years later by Little^4^ for human cells. Afterwards, the G_1_-checkpoint had somewhat fallen into oblivion as in many tumour cell lines it was not observed, which Kastan’s discovery explained by the fact that it required functionality of p53, often lost at later stages of tumour development. Although the capability to halt cell cycle progression for a few hours after irradiation should give cells additional time for repair before entry into the S-phase, a functional p53 does not necessarily convey greater radioresistence.^5^ Other factors, such as the checkpoint control in later phases of the cell cycle, clearly also play a role. There is, however, a clear advantage of cells capable of a G_1_-block in terms of how they proceed through the following S-phase: after a few hours of extra repair time cells have no problems completing replication of their previously damaged DNA, whereas cells incapable of such a halt tend to fail at some point during replication.^6^

Historically, greater attention has been given to the G_2_-checkpoint. It was observed first by Howard and Pelc^3^ in plant cells, and a decade later by Terasima and Tolmach^7^ in human cells. As for a possible role of p53 in controlling this checkpoint, the reports in the literature are somewhat contradictory. Cells deficient in p53 are capable of arresting cell cycle progression before entry into mitosis.^1^ We have demonstrated recently that while the accumulation of cells in the G_2_-compartment after irradiation can be different in p53 functional and non-functional tumour cell lines, the delay of the G_2_-phase itself is completely independent of the p53 status.^8^ Some authors have claimed, however, that manipulation of the p53 expression can have an effect on the G_2_-checkpoint.^9^,^10^ Others have suggested that p53 plays a role in the maintenance of the arrest 2 – 10 hours after radiation.^11^,^12^ It would seem, therefore, that both p53-dependent and p53-independent pathways play a role.^13^,^14^ Supposedly, the function of the G_2_-checkpoint is to allow for the repair of DNA damage before mitosis. This is in agreement with the observation that abrogation of the block, *e.g.* by high concentrations of caffeine, usually sensitizes cells to radiation^15^,^16^, although that does not seem to be the case with all cell types.^17^ Again, the interplay of different checkpoints may be of relevance here. Interestingly, although the length of the G_2_-phase delay did not correlate with radiation sensitivity as such, the number of unrejoined chromosome breaks was significantly elevated if the checkpoint was attenuated.^18^

And finally, regarding the role of p53 in the regulation of S-phase, there does not seem to be a great number of studies directly addressing the issue.^19^ Radiation induced delays in the S-phase have been described and their regulation has been analysed since Painter and Young discovered radioresistant DNA synthesis in Ataxia telangiectasia cells in 1980^20^, but it was not until a decade and a half later that authors began to speak of an S-phase damage checkpoint.^21^,^22^ This checkpoint seemed to be independent of p53. At least in some cases, however, p53 apparently did have an influence on how and when cells proceeded through replication (see below). Thus, the role of p53 in the regulation of the S-phase checkpoint “seems much more elusive” than its role for the G_1_- and G_2_-phases and “the details … are awaiting future studies”, as Fei and El-Deiry stated in their review 2003.^23^ That is still true 10 years later.

In general, when one looks at the regulation of S-phase after DNA damage, a rather complex, sometimes confusing picture presents itself. Two checkpoints can be distinguished, one involving stalled replication forks (called the “replication checkpoint”) and another activated by double strand breaks (called the “intra-S-phase checkpoint”).^24^,^25^ Replication forks can be stalled because of nucleotide starvation or because of inhibition of key replication enzymes, but also by certain types of DNA damage. In all of these cases, patches of single-stranded DNA appear to be the key signal, causing first the activation of ATR, which in turn leads to the phosphorylation of Dbf4 (directly) and Cdc7 (indirectly via activation of Chk1). The thus modified Cdc7/Dbf4 complex is now unable to initiate replication at hitherto unfired origins, but at the same time it protects the integrity of the stalled replication fork.^26^ Part of this process, namely the phosphorylation of Dbf4, can also be initiated by ATM, which is activated as a consequence of double strand break induction. Dbf4 is thus at the same time one of the components of the “replication checkpoint” and the “intra-S-phase checkpoint”, the latter being also under the influence of three more pathways.^27^–^29^ The first of these leads from ATM activation to the phosporylation of Smc1^30^ and Smc3^31^, associated proteins responsible for the maintenance of chromatin structure. The other two depend on the activation of either Chk1 (by ATR) or Chk2 (by ATM). Both of these checkpoint kinases can either directly phosphorylate Cdc25A, causing its degradation and thus preventing activation of Cdk2, which in turn blocks the formation of the pre-replication complexes and the firing of new origins.^32^,^33^ Or they can work through p53 which has a number of ways to influence replication: it causes the activation of killin, a nuclear inhibitor of DNA replication^34^,^35^; it has an influence on Cdc25A through a factor called ATF3, described as a transcriptional repressor^36^; it represses the transcription of Cdc25A through p21^37^, and finally it inhibits Cdk2 through p21 directly.^38^

In order to clarify how irradiated cells progress through the S-phase in the presence or absence of a functional p53, we compared 6 human tumour cell lines that had earlier been characterized as to their p53 status, their capability to control the G_1_-checkpoint and their tendency to fail during S-phase when irradiated in G_1_. We labelled cells in S-phase with the help of BrdU and followed their movement through the S- and G_2_-phases using a pulse-chase protocol. Clear differences between p53 functional and non-functional cells became obvious in this study, which suggest that the importance of p53 protein for the intra-S checkpoint has to be re-evaluated.

## Materials and methods

### Cell lines

The following human tumour cell lines were used^6^:

– Be11: A human melanoma cell line originally isolated in Dr. Malaise’s laboratory at the Institute Gustave-Roussy, Villejuif, France^39^; the cells have a DNA index of 1.6 and are only slightly pigmented.
– MeWo: A human melanoma cell line originally isolated by Dr. Fogh’s group in the Sloan-Kettering Institute for Cancer Research, New York^40^; the subline used in our institute has a DNA index of 1.6 and is no longer pigmented.
– 4197: A squamous carcinoma cell line derived from a tumour in the lower jaw of a 55-year-old male patient; it was established in 1987 from a biopsy taken at the department of maxillofacial surgery at the University Clinics in Essen; the cells have a DNA index of 1.0.
– 4451: A squamous carcinoma cell line derived from a recurrent tumour in the lower jaw of a 46-year-old male patient; it was established in 1988 from a biopsy taken at the aforementioned department; the cells have a DNA index of 1.5.
– EA14: A human malignant glioma cell line isolated in the Department of Radiotherapy at the University Clinics Essen 45; the cells have a DNA index of 1.3.
– U87: A human malignant glioma cell line isolated by Ponten and Macintyre at the Wallenberg Laboratory, Uppsala, Sweden; the cells have a DNA index of 1.0.

All six cell lines have been characterized with respect to their p53 status. In our own study of the first four^41^, we used a number of indirect methods suggesting that Be11 and 4197 were p53 wild types, but MeWo and 4451 were p53 mutants.^41^ Since then, MeWo and 4451 have been confirmed as mutants by direct DNA sequencing.^42^,^43^ The observation that Be11 and 4197 are p53 wild-types has been corroborated by analysis of their p21 expression after radiaton exposure, which is intact.^44^ The latter two cell lines have been studied by others.^46^ Both of them were reported to have a p53 wild-type gene sequence. However, a strong increase of p53 and p21 expression after irradiation was observed only in EA14, whereas U87 showed a much reduced increase in p53 and no increase at all in p21. We therefore designated Be11, 4197, and EA14 as p53 functional, but MeWo, 4451, and U87 as p53 non-functional.

### Culture conditions and treatment

The cells were routinely cultured in Minimal Essential Medium with Eagle’s salts, supplemented with 20% fetal calf serum. They were subcultured twice a week and routinely checked for mycoplasma contamination. For the experiments, cells from an exponentially growing culture were seeded into small culture flasks (25 m^2^, 5 ml medium, 250,000 cells). After 24 h, they were exposed to X-rays (Stabilipan‘ Siemens, 240kV, 0.5mm Cu filter, 15mA, 1Gy/min). All culture flasks, including sham irradiated controls, were taken from the incubator at the same time and kept together while successively treated in the adjacent room.

### Two-parameter flow cytometry^47^

Immediately after radiation exposure, 50μl of bromodeoxyuridine (BrdU) solution (1mM) was added to the flasks (final concentration 10μM), and the flasks were incubated for 30 min. The medium was then removed, the flasks washed twice, and the cells were further incubated in the absence of BrdU. Cells were trypsinized at 2 hour intervals (up to 10 hours after irradiation) and fixed in 96% ethanol.

The immunofluorescence staining for flow cytometry analysis has been described in detail elsewhere.^48^ Briefly, cells were incubated in a pepsin solution to isolate nuclei and then in 2 N HCl to partly denature the DNA. They were then incubated with anti-BrdU mouse IgG (Becton Dickinson, 1:20) followed by goat anti-mouse IgG FITC-conjugate (DuPont, 1:100). The DNA was stained with propidium iodide (PI). Green (FITC) and red (PI) fluorescence after 488-nm laser excitation were recorded with a FACScan flow cytometer (Becton Dickinson) and plotted in two-parameter scatter-grams. Ten thousand events were recorded; the coefficient of variation of the DNA histograms was about 5%.

### Data analysis

**FIGURE 1.**
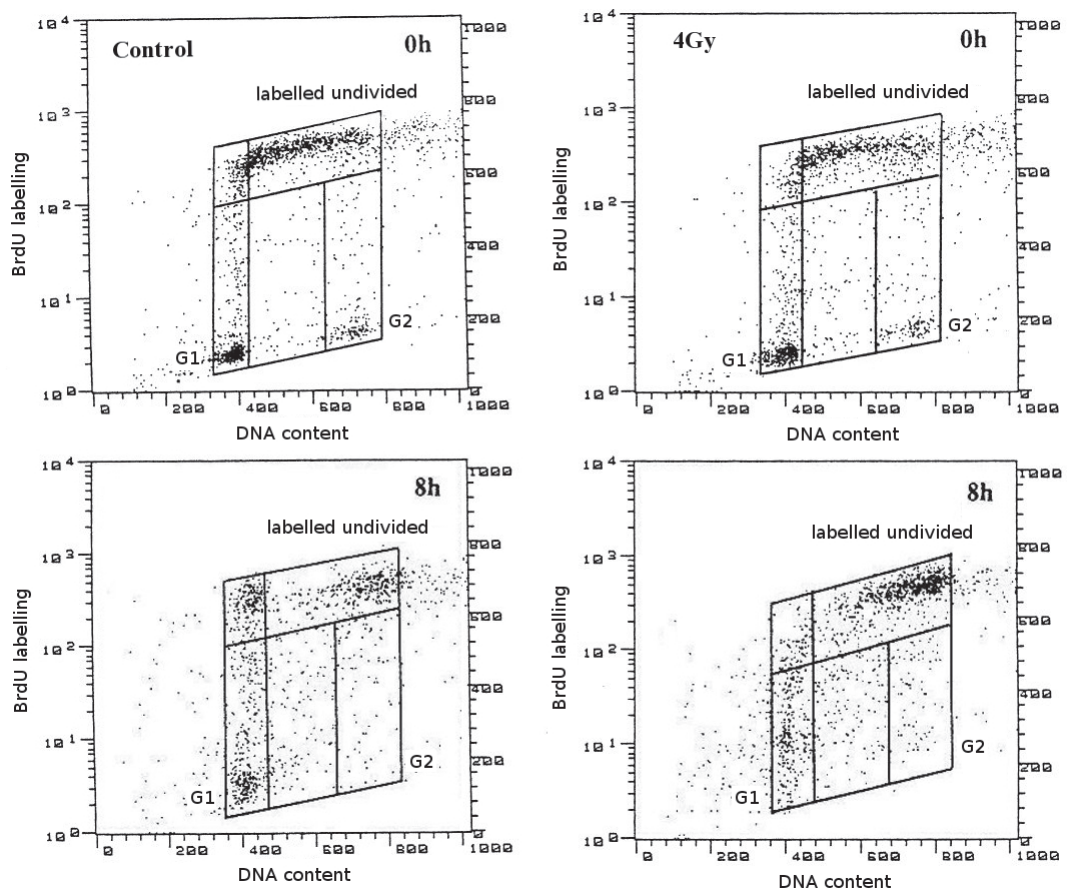
Examples of scattergrams of control cultures and cultures exposed to 4 Gy of X-rays, labelled with BrdU and kept in BrdU-free medium for the times indicated (MeWo) (FL1-H: BrdU incorporation (FITC), FL3-H: DNA content (PI))

Relative movement (RM) values were calculated as the mean DNA fluorescence of the BrdU labelled undivided cells (those that had not yet passed through mitosis, Figure 1) less the DNA fluorescence of the cells in G_1_ divided by the difference in DNA fluorescence of the cells in G_1_and G_2_^47^, ^49^:

$$\text{RM}=\frac{{\text{F}}_{\text{lab}}-{\text{F}}_{\text{G}1}}{{\text{F}}_{\text{G}2}-{\text{F}}_{\text{G}1}}$$

Each experiment was carried out four times with each cell line. All values given below are means and standard errors of the mean from interexperimental variation.

## Results

**FIGURE 2.**
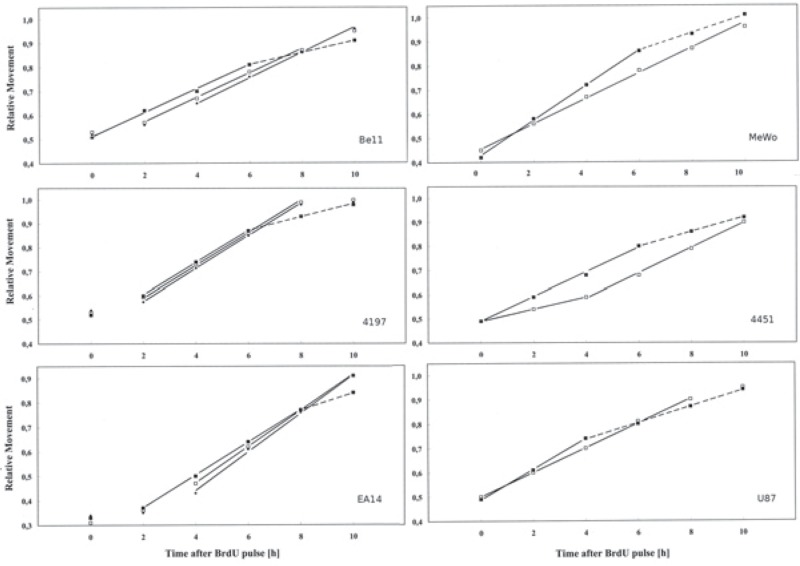
”Relative Movement“ as a function of time after radiation exposure in p53 functional (Be11, 4197, EA14) and p53 non-functional (MeWo, 4451, U97) cell lines. Error bars omitted for clarity. See Table 1 for means and standard errors of the mean from interexperimental variation. The extensions of the solid lines indicate which data points were included in the regression analysis. The broken lines depict the second, shallower component of the ”Relative Movement“ curves (see text).

The pulse-chase method which we employed here allowed us to follow the progression of labelled cells through S-phase in the course of several hours after irradiation. At the time of radiation exposure, S-phase cells were positioned, on average, at about half the distance between the G_1_ and G_2_-peaks. They then moved towards the G_2_-peak and after a few hours, having undergone mitosis, began to reappear in G_1_. Earlier research has shown that when the Relative Movement (the mean position of labelled cells between the G_1_ and G_2_-peaks) is plotted against time after labelling, it initially increases with a slope of 1/T_S_, where T_S_ is the duration of S-phase; when the first cells have passed through mitosis, the slope is reduced by a factor of 2.^47^,^49^ As shown in Figure 2, this was the case for all cell lines in the absence of irradiation. The “break-point” in the curves occurred mostly around 6 h, which is in reasonable agreement with estimates for their transit time through the G_2_-and M-phases.^8^,^50^ From the increase of the Relative Movement in the first hours after labelling we obtained values for the duration of the unperturbed S-phase between 14 h (MeWo) and 20 h (Be11). Table 1 suggests that there was no obvious connection with the p53 status.

Important differences between p53 functional and non-functional cells were seen, however, after irradiation. In the p53 functional cells, progression through the S-phase was halted for just a short time and then proceeded without further delay or even at a slightly accelerated pace. An initial delay was also seen, for 4197 and EA14, in unirradiated cells, but it was prolonged after radiation exposure. In p53 non-functional cells, on the other hand, progression through the S-phase was significantly slowed down over at least a few hours. In the case of 4451, the slow-down was particularly dramatic, but progression returned to normal after 4 h.

Within the first two pairs of cell lines, the p53 proficient ones were more radioresistant in terms of cell survival, so that it seemed advisable to also employ higher doses with them as compared to the p53 deficient ones (6 Gy for Be11, 8 Gy for 4197 leading to the same cell survival as 4 Gy in MeWo and 4415, resp.^41^). Not even with these higher doses, however, was their progression through the S-phase (after the initial short delay) significantly reduced. The same was the case with EA14 (where we also applied 8 Gy in spite of it being more radiosensitive than its counterpart U87^46^). We therefore concluded that the absence of a strong radiation effect on S-phase progression in the p53 functional cell lines was real and not due to their different radiosensitivity as compared to the p53 non-functional cell lines.

## Discussion

It must be emphasized that any conclusion that may be drawn here about the progression through S-phase in relation to p53 function, is restricted to cells which are in S-phase at the time of irradiation. This is due to the fact that labelling was done immediately after radiation exposure. For MeWo, we have some data from an earlier study where we looked at the Relative Movement of cells labelled 24 or 48 h after irradiation and we could still see a significant slow-down in S-phase at these times, although the G_2_-block had been completely overcome.^47^ No such experiments have been undertaken for any of the other cell lines used in the present investigation.

For cells in S-phase, then, it is clear that a functional p53 suppresses further DNA synthesis for no more than a short period, but permits progression through the S-phase at a normal pace afterwards. In cells that are p53 non-functional, DNA synthesis is slowed down for at least a couple of hours, in two of our three cell lines for the whole duration of the S-phase. Whether the p53 effect is on replicon initiation or on elongation in already initiated replicons is impossible to tell from our data. A complete halt of replication for 1–2 hours in p53 proficient cell lines would suggest that both are affected, but with up to 100 000 origins of replication in a human cell one would probably not notice if elongation in a few already initiated replicons was finished and just the firing of new origins prevented. Indeed, experiments in which the direct block of viral origin by p53 binding was studied after γ-irradiation, it was found that initiation was completely shut down, but elongation continued unabated even though the template must still have been damaged.^51^ This agrees with the conclusion of a much earlier study.^20^

On the other hand, an investigation of the different effects of irradiation on DNA synthesis in normal and Li-Fraumeni fibroblasts clearly showed that in the absence of p53 both initiation and elongation were slowed down.^52^ Although the experiments described in that study focused on overall DNA synthesis and were not designed to distinguish between a slow-down of progression through S-phase and a reduced entry of cells into S-phase, the findings reported seem to be in very good agreement with our data. In normal cells, DNA synthesis was shut down within an hour or two after irradiation, then recovered, and again decreased after about 6 hours. The latter effect was ascribed to less cells entering S-phase because of the G_1_-block. In p53-deficient fibroblasts, the initial drop in DNA synthesis was also present, but it lasted longer then in normal cells, recovered more fully and was not followed by a dramatic drop at later times, presumably because cells were not blocked in G_1_. Importantly, normal fibroblasts seemed to shut down only initiation, while both initiation and elongation were affected in p53-deficient cells.^52^

By which mechanism p53 suppresses replicon initiation and by which means both replicon initiation and elongation are suppressed in p53-deficient cells remains to be elucidated. In the introduction, we have mentioned a number of pathways involved in the intra-S-delay, but it was beyond our possibilities to examine the details of replication control in our cell lines. Nevertheless, the experiments presented here show that p53 does play a role for the progress of cells through the S-phase after X-ray exposure, at least with cells irradiated in S-phase itself.

## Figures and Tables

**TABLE 1.** Duration of S-phase and delay per unit dose calculated from data shown in Figure 2

**Cell line**	**Dose [Gy]**	**Duration of S-phase [h]**			**Delay [h/Gy] ^c^**
Be11	0			20.4 ±1.6 ^b^	
	4	0.8 ± 0.6 ^a^	+	19.8 ± 2.0 ^b^	− 0.15 ± 0.27
	6	1.2 ± 0.6 ^a^	+	19.4 ± 2.1 ^b^	− 0.17 ± 0.18
MeWo	0			13.7 ± 1.1 ^b^	
	4			17.5 ± 0.9 ^b^	0.95 ± 0.35
4197	0	0.8 ± 0.4 ^a^	+	15.4 ± 2.6 ^b^	
	4	1.0 ± 0.6 ^a^	+	15.1 ± 2.4 ^b^	− 0.08 ± 0.12
	8	1.2 ± 0.8 ^a^	+	14.8 ± 1.7 ^b^	− 0.07 ± 0.08
4451	0			19.6 ± 1.2 ^b^	
	4	(0 – 4 h)		40.7 ± 4.5 ^b^	5.28 ± 1.36
		(6 – 10 h)		19.2 ± 2.2 ^b^	− 0.10 ± 0.23
EA14	0	1.4 ± 0.5 ^a^	+	14.9 ± 0.4 ^b^	
	4	1.9 ± 1.0 ^a^	+	13.6 ± 1.5 ^b^	− 0.33 ± 0.19
	8	2.6 ± 0.4 ^a^	+	12.6 ± 0.5 ^b^	− 0.29 ± 0.09
U87	0			16.0 + 1.6 ^b^	
	4			19.6 ± 1.1 ^b^	0.90 ± 0.58

a The smaller figures are the initial lags immediately after irradiation.

b The larger figures are the durations of S-phase calculated from the slopes m of the “Relative Movement” curves (T_S_ = 1/m)

c Delays calculated from the slopes m of the “Relative Movement” curves (T_S_ = 1/m), i.e. neglecting the initial lags
